# Supplementary figures and images for: Estimating species sensitivity distributions on the basis of readily obtainable descriptors and toxicity data for three species of algae, crustaceans, and fish
Source: PeerJ. 2021 Mar 3;9:e10981. doi: 10.7717/peerj.10981 (PMC7936562; doi:10.7717/peerj.10981)

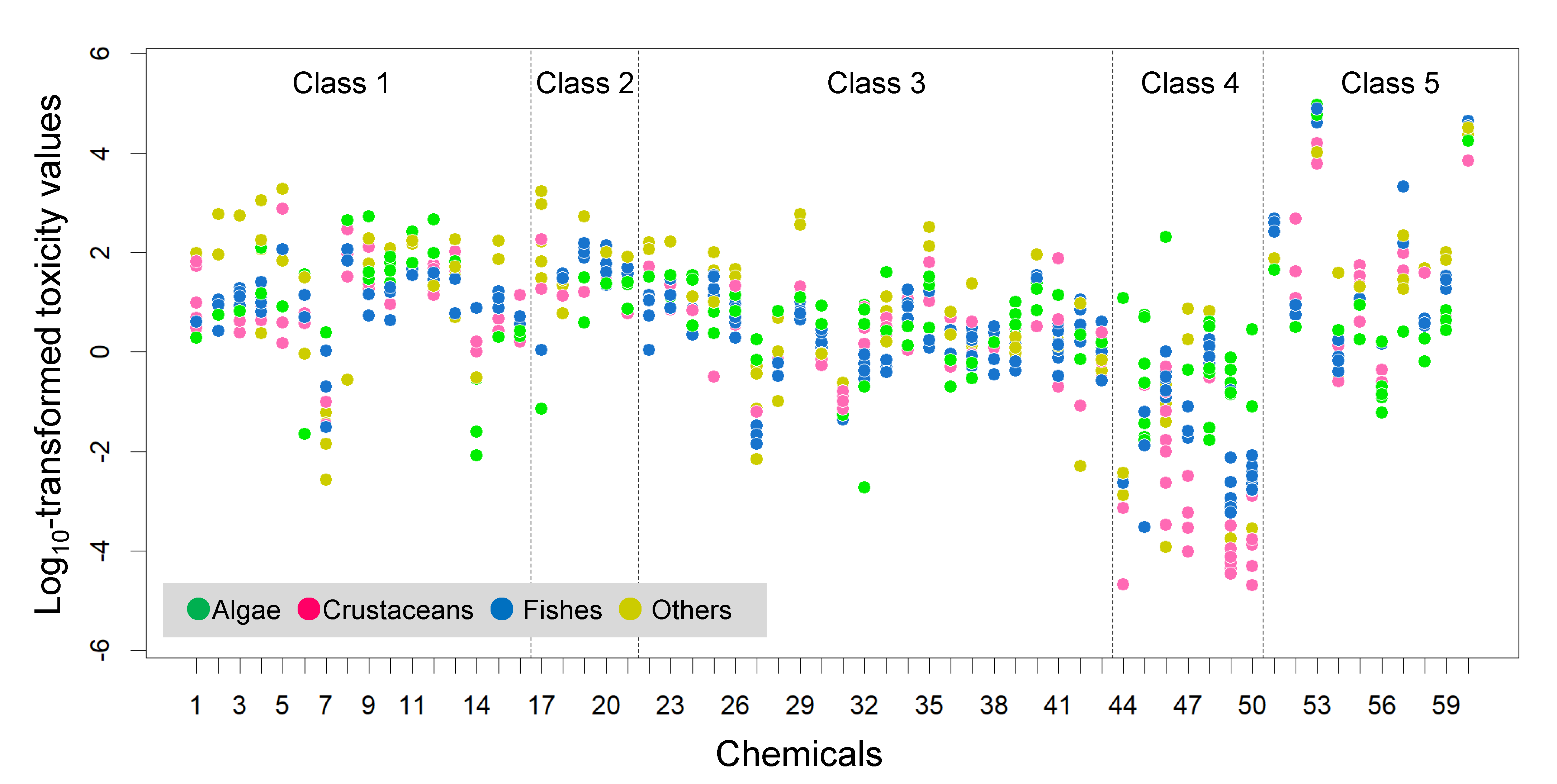

Supplement: Figure S1 — Out of seven Class 4 chemicals, five were used as insecticides; the other two were a fungicide and a herbicide. [file peerj-09-10981-s001.png]

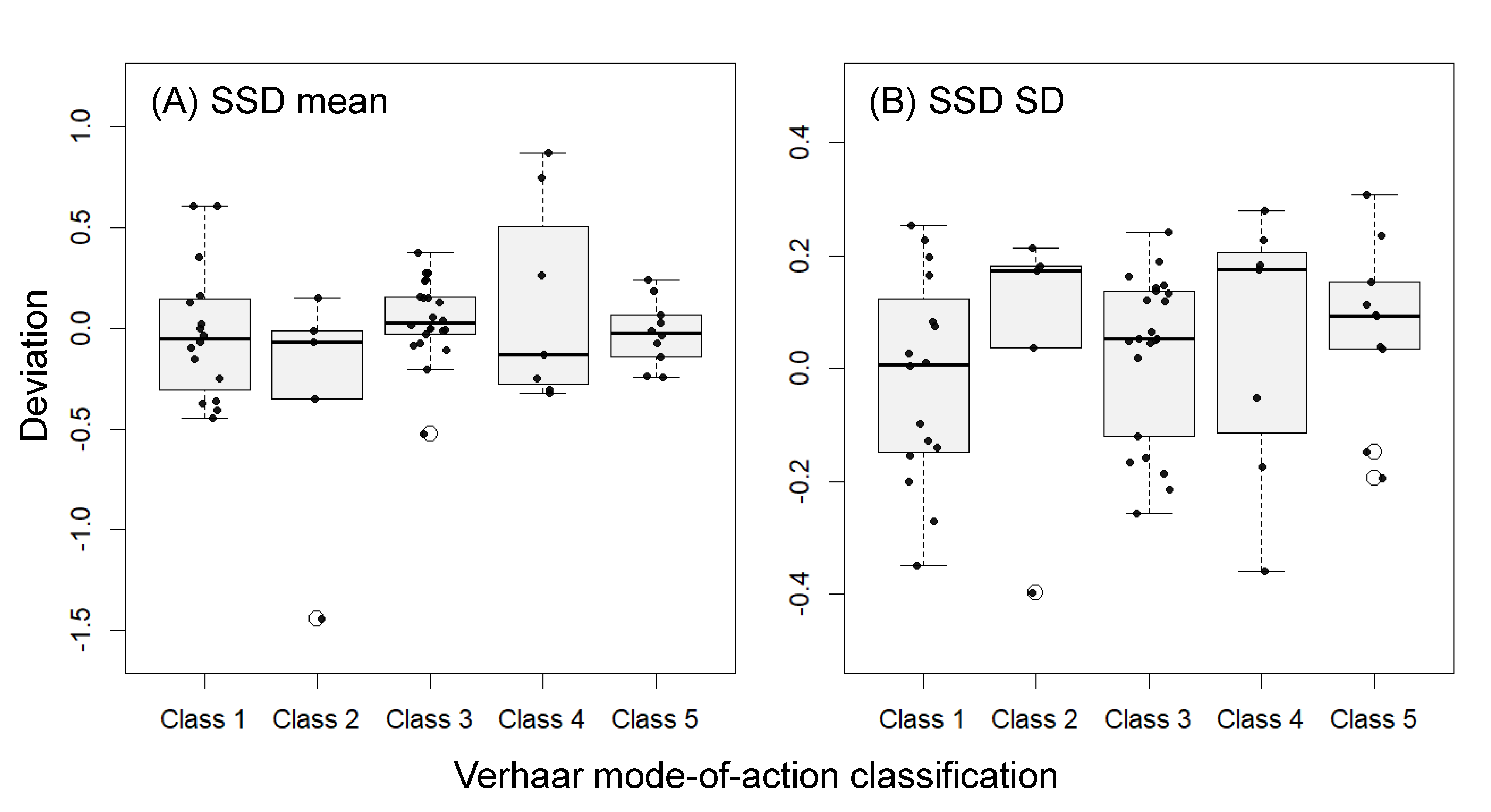

Supplement: Figure S2 — The predicted values are based on the best models with 3 species mean/SD and descriptors (see text). The bold horizontal lines, boxes, error bars, and white circles in the boxplots indicate the medians, interquartile ranges, 1.5× (interquartile ranges), and outliers, respectively. Black dots indicate individual values. [file peerj-09-10981-s002.png]
